# Development and Validation of a Standardized Lip Fullness Grading Scale

**DOI:** 10.1111/jocd.70773

**Published:** 2026-03-19

**Authors:** Sun Young Choi, Min Kyung Jeong, DaHye Jung, Beom Joon Kim

**Affiliations:** ^1^ Department of Dermatology Chung‐Ang University Gwangmyeong Hospital, Chung‐Ang University College of Medicine Gwangmyeong‐si Republic of Korea; ^2^ Department of Clinical Trial Huons Co. Ltd. Seongnam‐si Republic of Korea; ^3^ Department of Dermatology Chung‐Ang University Hospital, Chung‐Ang University College of Medicine Seoul Republic of Korea

**Keywords:** lip fullness scale, reliability, validation

## Abstract

**Background:**

Lip augmentation is a common cosmetic procedure that requires an objective and validated scale to assess treatment outcomes. Existing lip fullness scales may not be universally applicable across ethnic groups. This study aimed to develop and validate the Humedix Lip Fullness Scale and assess its agreement with the Allergan Lip Fullness Scale.

**Methods:**

A total of 53 females were photographed under standardized conditions. Five dermatologists independently evaluated lip fullness using the Humedix scale and analyzed intra‐ and inter‐rater reliability using Cohen's and Fleiss' Kappa, respectively. The same evaluators reassessed the images using the Allergan scale after 6 months, and agreement between the scales was analyzed using Cohen's Kappa.

**Results:**

Intra‐rater reliability was perfect (Cohen's Kappa = 1.0000), and inter‐rater reliability was high (0.9682, 95% confidence interval: 0.9377–0.9987). Agreement between the Humedix and Allergan scales was strong (Cohen's Kappa = 0.8456, weighted Kappa = 0.9056).

**Conclusion:**

The Humedix Lip Fullness Scale demonstrated excellent reliability and strong agreement with the Allergan scale, validating its use as an objective tool for assessing lip volume.

## 1 Introduction

Lip augmentation is an increasingly popular cosmetic procedure driven by the desire for fuller, more aesthetically defined lips. Various techniques of lip augmentation, both temporary and permanent, have been developed to cater to patient preferences and can be divided into two categories: injectable treatments and surgical procedures. Dermal filler injections are the most common method for enhancing lip size and volume. Other techniques include fat transfer, lip implants, lip lifts, lip threading, and laser lip plumping [1, 2].

Choosing the appropriate procedure requires careful consideration of individual goals, anatomy, and associated risks and benefits, given the diverse options. Optimizing techniques ensures efficacy and patient satisfaction. A precise and validated scale is essential for accurately assessing lip volume and treatment outcomes [3].

Several validated scales for assessing lip fullness have been developed. The Lip Fullness Grading Scale, a 5‐point photonumeric rating scale introduced in 2008, objectively quantifies three‐dimensional lip fullness [4]. The Medicis Lip Fullness Scale (introduced in 2012) [5] and the Allergan Lip Fullness Scale (developed in 2015) both evaluate upper and lower lip volume using separate 5‐point scales, with the latter validated for detecting clinically significant changes [6]. These scales are widely used in clinical research and practice to objectively measure changes in lip volume. Morphological differences in lip anatomy across ethnic groups are well‐documented [7, 8]. Many existing scales were developed and validated predominantly in Western populations, potentially limiting their generalizability in Asian patients or those with Fitzpatrick skin types III–VI. The Humedix Lip Fullness Scale was designed to reflect these morphological variations and enhance representation of lip characteristics specific to Asian populations.

This study aims to develop and validate a lip fullness scale. Furthermore, it seeks to compare the agreement between the newly developed Humedix Lip Fullness Scale and the Allergan Lip Fullness Scale.

## 2 Materials and Methods

### 2.1 Target Participants and Collection of Photographs

Overall, 53 healthy adults (≥ 20 years) were recruited for lip fullness photography and data collection. Eligibility criteria include healthy adults aged ≥ 20 years and the absence of acute or chronic diseases, including infectious skin conditions. Participants provided informed consent after a thorough explanation from the investigator. The study was approved by the Institutional Review Board of P&K Skin Research Center (IRB No. P2311‐5216). Exclusion criteria included pregnancy, breastfeeding, or potential pregnancy; refusal to participate or sign the consent form; psychiatric disorders; those who received immunosuppressive therapy within the past 3 months or systemic steroids or phototherapy in the past month; lesions in the test area interfering with measurements; severe atopic dermatitis, infectious skin diseases, or severe allergies or reactions to cosmetics, medications, or daily UV exposure; and skin treatments, including scaling, within the last 3 months. Eligible participants underwent lip fullness photography.

Photographs were taken from three angles—front, left lateral, and right lateral—using a Canon EOS 850D camera (Japan) under consistent lighting. Camera settings were standardized (shutter speed: 1/60, aperture: F8, ISO: 1600). A single photographer took all photographs. Participants were instructed to remove their makeup, sit in an upright position on a chair, and face the camera directly with their eyes closed. The positions (front, left, and right) were pre‐marked on the floor and chair. The camera was placed on a tripod to prevent shaking during the shoot.

### 2.2 Validation of Assessment Scale

The Humedix Lip Fullness Scale is a 5‐point photonumeric grading system: Grade 0 (minimal)—flat or nearly flat contour with minimal visible red lip; Grade 1 (mild)—some red lip visible; no lower lip pout; Grade 2 (moderate)—moderate red lip visible with slight lower lip pout; Grade 3 (marked)—significant red lip visible with lower lip pout and moderate upper lip protrusion; and Grade 4 (very marked)—very significant red lip visible with pronounced lower lip pout and overall lip protrusion.

A set of 53 photographs was randomly arranged and evaluated by five dermatologists. To eliminate timing bias, photos were randomized without regard to capture time. Each evaluator independently assessed the photographs without exchanging opinions or discussing the results. All photographs were reviewed on the same computer monitor, and grades were recorded individually. Lip fullness was determined based on agreement among at least four evaluators, while unanimous selections were used as representative images for each grade. Validation analysis assessed inter‐ and intra‐rater reliability using the Kappa coefficient.

### 2.3 Comparison of Lip Fullness Grades Scales

The photographs of the 53 participants, previously evaluated using the Humedix Lip Fullness Scale, were re‐evaluated by the same dermatologists using the Allergan Lip Fullness Scale (Table 1) to assess scale agreement. The re‐evaluation occurred approximately 6 months later, with the photographs presented in a randomized order. To ensure proper blinding, the lip photographs were re‐randomized and relabeled with new anonymous identifiers prior to the 6‐month reassessment. Evaluators were blinded to both participant identity and their own previous ratings. All evaluation materials were distributed and collected independently to maintain full separation and avoid potential bias.

**TABLE 1:** The Allergan Lip Fullness Scale.

Score	Grade	Definition
0	Minimal	Flat or nearly flat contour; minimal red lip shows.
1	Mild	Some red lips shows; no lower lip pout.
2	Moderate	Moderate red lip shows with slight lower lip pout.
3	Marked	Significant red lip shows and lower lip pout; upper lip with moderate pout.
4	Very marked	Very significant red lip shows, lower lip pout, and upper lip pout.

The front, right‐side, and left‐side photographs of each participant were randomized regardless of grade. Five evaluators reviewed and assessed these photographs on the same computer monitor based on the Allergan Lip Fullness Scale and assigned a single grade for each participant. The final grade for each participant was based on agreement among at least four evaluators. Lip fullness grades from the Allergan and Humedix scales were then compared.

### 2.4 Statistical Analysis

Statistical analyses were performed using SAS (Version 9.4), with confidence intervals (CIs) and continuous data rounded to four decimal places. Statistical significance was set at a significance level of 0.05. The sample size of 53 participants was based on a power analysis using Walter's method [9], with assumptions of a minimal acceptable Kappa (*ρ*₀) of 0.49 [10], an expected Kappa (*ρ*₁) of 0.69 [11], *α* = 0.05, power = 90%, and seven raters. The required sample size was 37; after adjusting for a 22% potential drop‐out rate and adding five participants for scale development, the final sample was set at 53.

Fleiss' Kappa was used to assess inter‐rater agreement for the Humedix Lip Fullness Scale, while Cohen's Kappa was used to evaluate intra‐rater consistency. All participants were assessed via the same rater.

Cohen's Kappa was also used to measure the agreement between the Humedix and Allergan scales for categorical values [11]. The calculated Cohen's Kappa statistic was interpreted based on Altman's criteria, where values above 0.81 indicate very good agreement [10].

## 3 Results

Photographs of 53 female participants were collected, evaluated, and validated. Table 2 presents their demographic details.

**TABLE 2:** Participant demographics.

Characteristics		Number (%) (*N* = 53)
Age (years)	20–29	2 (3.77)
	30–39	3 (5.66)
	40–49	14 (26.42)
	50–59	29 (54.72)
	60–69	5 (9.43)
Race	Asian	51 (96.23)
	African American	2 (3.77)
Fitzpatrick skin phototype	III/IV	51 (96.23)
	V/VI	2 (3.77)

**FIGURE 1:**
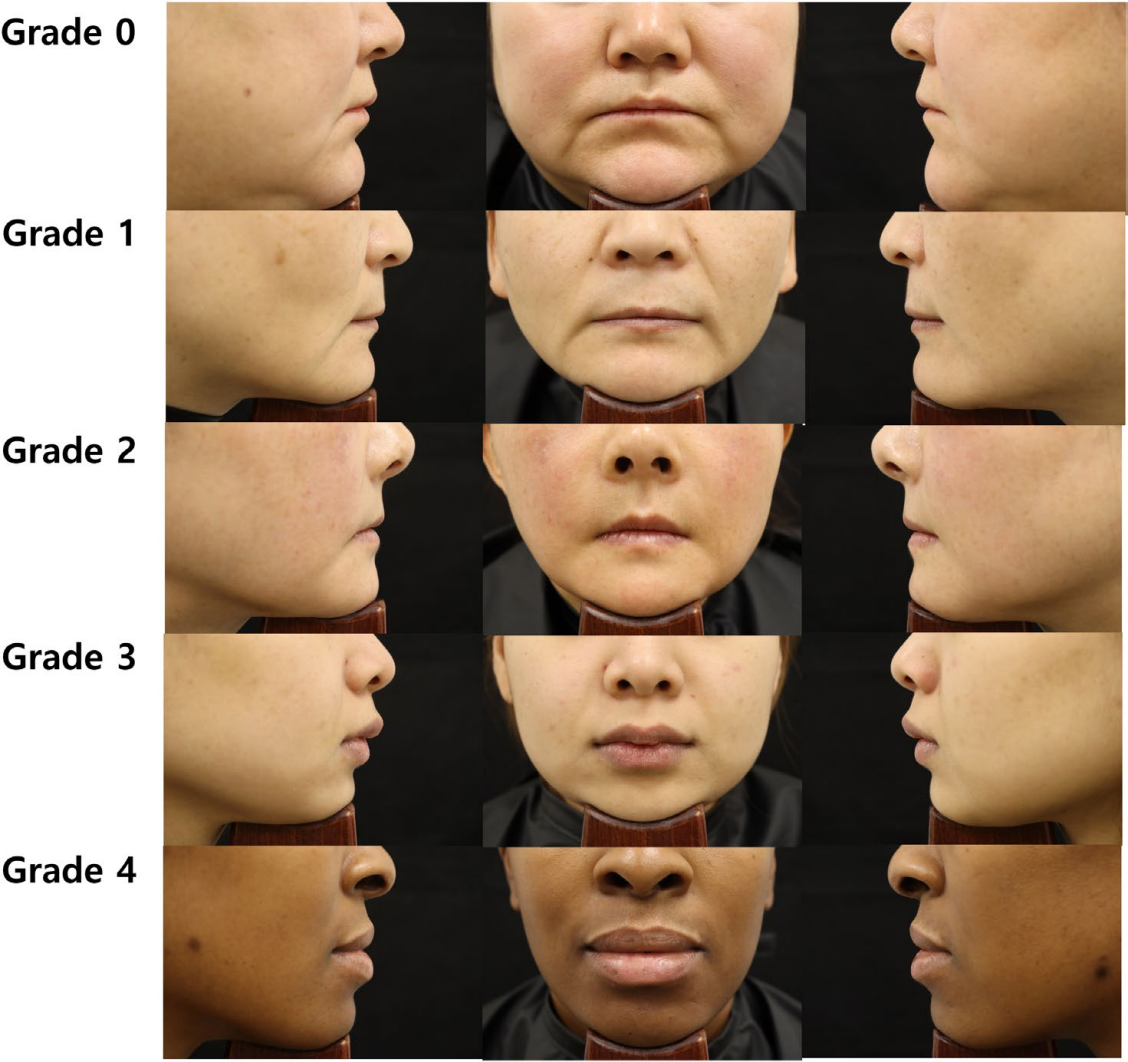
Photo‐guideline of the Humedix Lip Fullness Scale. Grade 0 (minimal): Flat or nearly flat contour with minimal red lip visible; Grade 1 (mild): Some red lip visible with no lower lip pout; Grade 2 (moderate): Moderate red lip is visible, with a slight lower lip pout; Grade 3 (marked): Significant red lip is visible, with a lower lip pout and moderate upper lip protrusion; and Grade 4 (very marked): Very significant red lip is visible, with pronounced lower lip pout and overall lip protrusion.

All five raters achieved perfect intra‐rater reliability (weighted Cohen's kappa = 1.0000), indicating completely reproducible assessments. The Fleiss' Kappa coefficient for inter‐rater agreement was 0.96821 (95% CI: 0.93771–0.99870), indicating near‐perfect consistency among raters (Table 3). This validates the Humedix Lip Fullness Scale as highly reliable for both intra‐ and inter‐rater assessments. Representative photographs for each grade of the Humedix Lip Fullness Scale are shown in Figure 1.

**TABLE 3:** Intra‐rater weighted kappa coefficients (Cohen's kappa) and overall inter‐rater kappa coefficients (Fleiss' kappa).

Investigator	Cohen's kappa	95% CI
1	1.0000	(1.0000, 1.0000)
2	1.0000	(1.0000, 1.0000)
3	1.0000	(1.0000, 1.0000)
4	1.0000	(1.0000, 1.0000)
5	1.0000	(1.0000, 1.0000)
	*Fleiss' kappa*	*95% CI*
Overall	0.96821	(0.93771–0.99870)

Abbreviation: CI, confidence interval.

The agreement between the Humedix and Allergan Lip Fullness Scales was evaluated using Cohen's Kappa statistic for all 53 participants. The cross assessment between the two scales is presented in Table 4. The initial evaluation was conducted using the Humedix Lip Fullness Scale, followed by a re‐evaluation using the Allergan Lip Fullness Scale. Cohen's Kappa was 0.8456 (95% CI: 0.7297–0.9616), and the weighted Cohen's Kappa was [10] 0.9056 (95% CI: 0.8333–0.9779), both exceeding 0.81, confirming a strong agreement between the scales.

**TABLE 4:** Cross table of the Humedix and Allergan Lip Fullness Scale assessment.

		Re‐assessment					Total
		Grade 0	Grade 1	Grade 2	Grade 3	Grade 4	
Initial assessment	Grade 0	3	2	0	0	0	5
	Grade 1	0	5	0	0	0	5
	Grade 2	0	0	12	1	0	13
	Grade 3	0	0	3	19	0	22
	Grade 4	0	0	0	0	8	8
Total		3	7	15	20	8	53

## 4 Discussion

The lips significantly influence facial aesthetics. Beyond lip volume, demand for cosmetic treatments addressing lip wrinkles, pigmentation, and related concerns is rising. Validated scales are crucial for accurately measuring these aesthetic outcomes, ensuring consistency in patient evaluations. Proper validation of these scales is also a prerequisite for designing and conducting clinical studies to help establish reliable endpoints for assessing treatment efficacy [3, 12].

Lip volume and shape vary widely by ethnicity, age, and sex. Individuals of African descent typically have fuller lips than those of European or East Asian descent, who generally have thinner lips due to genetic variations in facial structure and soft tissue [17]. These variations may affect lip fullness scale evaluations across different ethnic groups. Additionally, ethnicity and age should be considered when performing lip augmentation procedures, as they influence baseline lip characteristics and the desired aesthetic outcome [18].

To address these variations, our study primarily focused on Asian individuals to develop and validate the Humedix Lip Fullness Scale. We assessed its agreement using the widely used Allergan Lip Fullness Scale finding strong intra‐ and inter‐rater reliability. Five experienced dermatologists validated the scale, and when the Humedix and Allergan scales were applied to the same photographs, the agreement between the two scales remained high. Importantly, the Humedix Lip Fullness Scale was developed using an Asian‐majority dataset (96.2%), addressing the underrepresentation of this population in prior scale validations. The inclusion of Fitzpatrick skin types III–VI further enhances the scale's relevance across ethnicities. While this improves the Humedix Lip Fullness Scale applicability for Asian populations, the exclusive use of female subjects may limit generalizability. Future studies should validate the Humedix Lip Fullness Scale in male and multiethnic cohorts. Nonetheless, the Humedix Lip Fullness Scale offers clinicians a standardized and ethnically relevant tool for clinical practice, aesthetic evaluation, and outcome assessment in clinical trials.

In conclusion, the Humedix Lip Fullness Scale is a reliable tool for objectively evaluating lip volume and assessing treatment outcomes following various lip enhancement procedures. The Humedix Lip Fullness Scale can be incorporated into clinical trials evaluating dermal fillers or aesthetic interventions. It also provides a consistent and objective tool for monitoring patient outcomes in daily dermatologic and cosmetic practice.

## Funding

This work was funded by Humedix Co. Ltd., Seongnam‐si, Gyeonggi‐do, Republic of Korea.

## Ethics Statement

The study was approved by the institutional review board (IRB No. P2311‐5216). All participants consented to the reproduction and distribution of any images collected during the study.

## Conflicts of Interest

The authors declare no conflicts of interest.

## Data Availability

The data supporting the findings of this study are available from the corresponding author, KBJ, upon reasonable request.
